# Utilizing 3D fast spin echo anatomical imaging to reduce the number of contrast preparations in $T_{1\rho }$ quantification of knee cartilage using learning‐based methods

**DOI:** 10.1002/mrm.70022

**Published:** 2025-08-05

**Authors:** Junru Zhong, Chaoxing Huang, Ziqiang Yu, Fan Xiao, Thierry Blu, Siyue Li, Tim‐Yun Michael Ong, Ki‐Wai Kevin Ho, Queenie Chan, James F. Griffith, Weitian Chen

**Affiliations:** ^1^ CU Lab of AI in Radiology (CLAIR), Department of Imaging and Interventional Radiology The Chinese University of Hong Kong Hong Kong SAR China; ^2^ Department of Radiology Shanghai Sixth People's Hospital Affiliated to Shanghai Jiao Tong University School of Medicine Shanghai China; ^3^ Department of Electrical Engineering National Taiwan University Taipei Taiwan; ^4^ Department of Orthopaedics & Traumatology The Chinese University of Hong Kong Hong Kong SAR China; ^5^ Department of Orthopaedics & Traumatology The Chinese University of Hong Kong Medical Centre Hong Kong SAR China; ^6^ Philips Healthcare Hong Kong China

**Keywords:** $T_{1\rho }$ MRI, deep learning, osteoarthritis

## Abstract

**Purpose:**

To propose and evaluate an accelerated $T_{1\rho }$ quantification method that combines $T_{1\rho }$‐weighted fast spin echo (FSE) images and proton density (PD)‐weighted anatomical FSE images, leveraging deep learning models for $T_{1\rho }$ mapping. The goal is to reduce scan time and facilitate integration into routine clinical workflows for osteoarthritis (OA) assessment.

**Methods:**

This retrospective study utilized MRI data from 40 participants (30 OA patients and 10 healthy volunteers). A volume of PD‐weighted anatomical FSE images and a volume of $T_{1\rho }$‐weighted images acquired at a non‐zero spin‐lock time were used as input to train deep learning models, including a 2D U‐Net and a multi‐layer perceptron (MLP). $T_{1\rho }$ maps generated by these models were compared with ground truth maps derived from a traditional non‐linear least squares (NLLS) fitting method using four $T_{1\rho }$‐weighted images. Evaluation metrics included mean absolute error (MAE), mean absolute percentage error (MAPE), regional error (RE), and regional percentage error (RPE).

**Results:**

The best‐performed deep learning models achieved RPEs below 5% across all evaluated scenarios. This performance was consistent even in reduced acquisition settings that included only one PD‐weighted image and one $T_{1\rho }$‐weighted image, where NLLS methods cannot be applied. Furthermore, the results were comparable to those obtained with NLLS when longer acquisitions with four $T_{1\rho }$‐weighted images were used.

**Conclusion:**

The proposed approach enables efficient $T_{1\rho }$ mapping using PD‐weighted anatomical images, reducing scan time while maintaining clinical standards. This method has the potential to facilitate the integration of quantitative MRI techniques into routine clinical practice, benefiting OA diagnosis and monitoring.

## 1 INTRODUCTION

Spin‐lattice relaxation time in the rotating frame ($T_{1\rho }$) imaging is an advanced MRI technique for evaluating cartilage composition^1^, ^2^. Studies have shown that proteoglycan loss in cartilage is associated with increased $T_{1\rho }$ values^3^, ^4^. This method can be performed on standard 1.5T and 3T MRI scanners without requiring specialized hardware or contrast agents, making it a promising tool for the early detection of osteoarthritis (OA) and the monitoring of cartilage therapies in clinical practice. Extended scan time is a major challenge in $T_{1\rho }$ quantification. For example, the knee $T_{1\rho }$ imaging protocol recommended by the Radiological Society of North America (RSNA) requires approximately 6 to 12 min.^2^ This is because multiple $T_{1\rho }$‐weighted images must be acquired at the same location to fit a $T_{1\rho }$ relaxation model and calculate $T_{1\rho }$ maps. To address this, many studies have investigated methods to accelerate $T_{1\rho }$ imaging, such as k‐space undersampling or reducing the number of $T_{1\rho }$‐weighted images^5^, ^6^, ^7^, ^8^.

The two‐parameter mono‐exponential relaxation model is commonly used for $T_{1\rho }$ quantification. This model requires a minimum of two $T_{1\rho }$‐weighted images, though four images are often recommended to ensure robust quantification.^2^ Reducing the number of $T_{1\rho }$‐weighted images increases sensitivity to noise, necessitating a high signal‐to‐noise ratio (SNR) in the acquired data. Recent advances in deep learning have shown that reliable $T_{1\rho }$ mapping can be achieved using only two $T_{1\rho }$‐weighted images.^7^, ^8^ These methods leverage large training datasets and underlying signal models to enable robust $T_{1\rho }$ prediction. In this context, deep learning neural networks act as approximations of the $T_{1\rho }$ signal equation^9^ and prior research has demonstrated their effectiveness in predicting MRI parameters in scenarios where images exhibit relatively low SNR.^10^ Inspired by the representational capabilities of deep learning, we hypothesized that $T_{1\rho }$ imaging could be further accelerated through an optimized acquisition strategy. For instance, $T_{1\rho }$ could be predicted using just two images: One $T_{1\rho }$‐weighted image and one image acquired with a standard clinical pulse sequence.

In this study, we examined the feasibility of generating $T_{1\rho }$ maps using a proton‐density (PD)‐weighted anatomical image and a single $T_{1\rho }$‐weighted image, both acquired with fast/turbo spin echo (FSE/TSE) sequences. As there is no established signal model for deriving $T_{1\rho }$ from PD‐weighted images, such generation cannot be accomplished using the conventional non‐linear least‐squares (NLLS) fitting approach. Consequently, our experiment employed deep learning techniques to predict $T_{1\rho }$ values from a $T_{1\rho }$‐weighted image acquired with a $T_{1\rho }$‐prepared FSE sequence,^11^, ^12^ and a the PD‐weighted FSE acquisition served as a surrogate for image contrast corresponding to a time‐of‐spin‐lock (TSL) of zero in conventional quantitative $T_{1\rho }$ imaging. Additionally, we explored deep learning‐based $T_{1\rho }$ predictions using two $T_{1\rho }$‐weighted images. Our results demonstrated that the $T_{1\rho }$ predictions generated by our proposed methods were comparable to the $T_{1\rho }$ ground truth obtained by fitting four $T_{1\rho }$‐weighted images. Preliminary findings from this study were presented in an abstract at ISMRM 2025.^13^

## 2 METHODS

### 2.1 MRI pulse sequence

**FIGURE 1:**
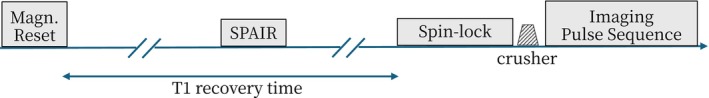
$T_{1\rho }$ pulse sequence diagram. magn. reset = magnetization reset.

Shown in Figure 1, we followed the standard framework for pulse sequence design commonly used in $T_{1\rho }$‐prepared pulse sequences for quantitative $T_{1\rho }$ imaging^12^, ^14^, ^15^, ^16^, ^17^ to collect the $T_{1\rho }$ maps utilized in this study. Specifically, we employed a magnetization‐prepared 3D FSE acquisition^12^, ^16^ to generate the $T_{1\rho }$ maps. The pulse sequence begins with a magnetization reset to saturate the magnetization,^14^ followed by a $T_{1}$ recovery period, a spin‐lock preparation module, and a 3D FSE acquisition. SPectral Attenuated Inversion Recovery (SPAIR) was implemented during the $T_{1}$ recovery period for fat suppression. The spin‐lock module consists of a 90‐degree tip‐down RF pulse to tip magnetization into the transverse plane, succeeded by spin‐lock RF pulse clusters with a duration TSL and amplitude at the frequency of spin‐lock (FSL), followed by a 90‐degree tip‐up RF pulse to flip magnetization to the longitudinal direction. The $T_{1\rho }$‐preparation was compensated for $B_{1}$ and $B_{0}$ using a 180‐degree pulse between the rotary echo as described by Witschey et al.^18^ Following the $T_{1\rho }$ preparation, imaging data were acquired using a vendor‐provided commercial 3D FSE sequence, VISTA^TM^ (Philips Healthcare, Best, Netherlands). Centric view ordering was used for phase encoding in k‐space.^19^ To minimize potential artifacts caused by rapid signal variations at the start of the FSE echo train,^19^ the phase encoding gradient was not activated for the first 4 echoes. The center of k‐space was acquired at the 5th echo. In this study, the time between the excitation RF pulse and the acquisition of the center of k‐space is referred to as TE. Note that this definition of TE differs from the contrast‐equivalent TE used in 3D FSE pulse sequences.^20^

With the aforementioned pulse sequence design, the magnetization due to $T_{1\rho }$ relaxation follows the signal equation below:

(1) $$I_{k}=I_{0}e^{-\frac{1}{T_{1\rho }}{\text{TSL}}_{k}}$$

where $I_{0}$ and $I_{k}$ are the magnitude of the $T_{1\rho }$‐weighted images acquired with TSL = 0 ms and TSL = TSL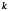, respectively.

The protocol of the PD‐weighted 3D FSE sequence was implemented using the commercially available VISTA^TM^ pulse sequence (Philips Healthcare, Best, Netherlands). Together with the aforementioned $T_{1\rho }$ pulse sequence, the detailed MRI parameters are available in Table 2.

It is noteworthy that with such a pulse sequence design, the $T_{1\rho }$‐weighted images acquired at TSL = 0 ms have contrast comparable to conventional PD‐weighted images. Consequently, we hypothesize that a 3D PD‐weighted anatomical FSE image, combined with a single $T_{1\rho }$‐weighted image acquired at a non‐zero TSL, can be utilized to achieve simultaneous $T_{1\rho }$ quantification and anatomical imaging.

### 2.2 Data acquisition

We retrospectively conducted our *in vivo* experiments on a previously reported dataset.^22^ Our study received approval from the institutional review board, and all participants provided informed consent. The dataset comprised 40 participants (30 OA patients and 10 healthy volunteers), with a mean age of 56.4 ± 19.9 years (mean ± standard deviation) and a mean body mass index (BMI) of 24.7 ± 4.2 kg/m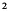 (mean ± standard deviation). 14 (35.00%) of the participants were male. We presented the detailed demographics for the OA patients and healthy volunteers separately in Table 1.

**TABLE 1:** Demographics.

	Patient (n = 30)	Healthy volunteer (n = 10)
Age (years)	67.63 ± 5.80	24.90 ± 2.59
BMI^a^ (kg/m 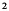 )	26.00 ± 4.08	22.75 ± 4.51
Male [*n* (%)]	9 (30.00)	5 (50.00)
Female [*n* (%)]	21 (70.00)	5 (50.00)
K‐L^b^ 0 [*n* (%)]	N/A^c^	10 (100.00)
K‐L 1 [*n* (%)]	2 (6.67)	N/A
K‐L 2 [*n* (%)]	13 (43.33)	N/A
K‐L 3 [*n* (%)]	6 (20.00)	N/A
K‐L 4 [*n* (%)]	9 (30.00)	N/A

*Note*: Age and BMI were reported in mean ± standard deviation.

^a^ BMI = body mass index.

^b^ K‐L = Kellgren‐Lawrence grades.^21^

^c^ N/A = Not Applicable.

For every participant, four 3D volumes of $T_{1\rho }$‐weighted images were acquired with an FSL of 300 Hz and TSL values of 0, 10, 30, 50ms, respectively, using a 3T clinical MRI scanner (Achieva, Philips Healthcare, Best, Netherlands). Additionally, we collected a 3D volume of PD‐weighted, fat‐suppressed anatomical FSE images. A single volume of $T_{1\rho }$‐weighted images was retrospectively selected, along with the volume of PD‐weighted anatomical images, to estimate the $T_{1\rho }$ map using the proposed learning‐based method. The $T_{1\rho }$ maps estimated from all four $T_{1\rho }$‐weighted images with TSL 0, 10, 30, 50ms using an NLLS fitting method were used as the ground truth. Detailed MRI acquisition parameters are provided in Table 2.

**TABLE 2:** MRI acquisition parameters.

Parameter	$T_{1\rho }$	PD‐weighted FSE
Plane	Sagittal	Sagittal
Fat suppression	SPAIR^a^	SPAIR
No. of slices	44	292
Field of view (${\text{mm}}^{3}$)	160 × 160 × 132	130 × 150 × 161
TE^b^/TR^c^ (ms)	31/2000	30/1200
Resolution (${\text{mm}}^{3}$)	0.8 × 1 × 3	0.55 × 0.545 × 0.55
Spin‐lock frequency (Hz)	300	N/A^d^
Spin‐lock time (ms, in acquisition order)	0/10/30/50	N/A
Scan time (min: sec)	4:02	7:20

^a^ SPAIR = SPectral Attenuated Inversion Recovery.

^b^ TE = echo time.

^c^ TR = repetition time.

^d^ N/A = Not Applicable.

### 2.3 $T_{1\rho }$ prediction

In this section, we introduce the deep learning‐based neural networks that predict $T_{1\rho }$ from the acquired data and accompanying preprocessing.

#### 2.3.1 Preprocessing

We segment the femoral, tibial, and patellar cartilage into one unified cartilage region of interest (ROI) on the $T_{1\rho }$‐weighted images. Concurrently, we registered the PD‐weighted FSE images with the $T_{1\rho }$‐weighted images. It is important to note that each pair of PD‐weighted and $T_{1\rho }$‐weighted images must be collected from the same subjects.

The registration process was executed in three stages: rigid, affine, and symmetric deformable with a validated method.^23^ Registration is crucial in the experimental framework due to the significant differences between the two sequences used for acquisition. Our registration method demonstrated a mean ± standard deviation of Structural Similarity Index of 0.97 ± 0.01 within the cartilage ROI. Detailed registration steps and performance calculations are provided in Supporting Information S1. In certain experiments, we further processed the data using ROI masks. Specifically, only the regions within the ROI were retained, while the voxels outside the ROI were set to 0. These experiments are detailed in Section 2.4.3.

#### 2.3.2 Deep learning models

We developed two neural network architectures specifically designed for this task: A 2D U‐Net with an output range limiter and a multi‐layer perceptron (MLP) model incorporating skip connections. To compare the efficacy of these two models, we conducted experiments as described in Section 2.4.2.

##### 2D U‐Net architecture

**FIGURE 2:**
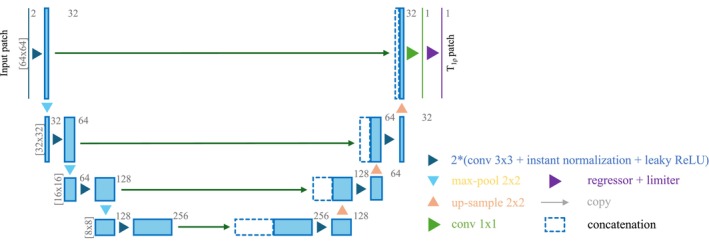
Architecture of the 2D U‐Net model. conv = convolution layer.

Figure 2 illustrates the architecture of the 2D U‐Net model structure. We adopted a standard U‐Net structure^24^ with modifications to accommodate our $T_{1\rho }$ prediction task by incorporating a regressor and a limiter. The regressor generated a continuous $T_{1\rho }$ prediction, while the limiter constrains the gradient of the mispredicted $T_{1\rho }$ value.

The limiter first applied ReLU activation^25^ to eliminate the negative inputs. Subsequently, we biased the ReLU output with the minimum value and clamped the final output. We formulate this limiter in Equation (2).

(2) $$ŷ=\{y_{min},\mathrm{ReLU}(x)+y_{min},y_{max}\}$$

where ŷ represents the final prediction, x denotes the output of the regressor, and $y_{min}$ and $y_{max}$ are hyperparameters to regulate the range of the prediction. These values were set to 10 and 100 based on our prior experience. By incorporating domain knowledge through the limiter, we facilitate more rapid convergence.

The 2D U‐Net model was trained using the ground truth $T_{1\rho }$ maps and an L1 (mean absolute error, MAE) loss function. Training occurred over 1 000 epochs using the Adam optimizer^26^ with an initial learning rate of 0.001, which was exponentially decayed by a factor of 0.9 as the training progressed. The weight decay was set to 0.0003.

**FIGURE 3:**
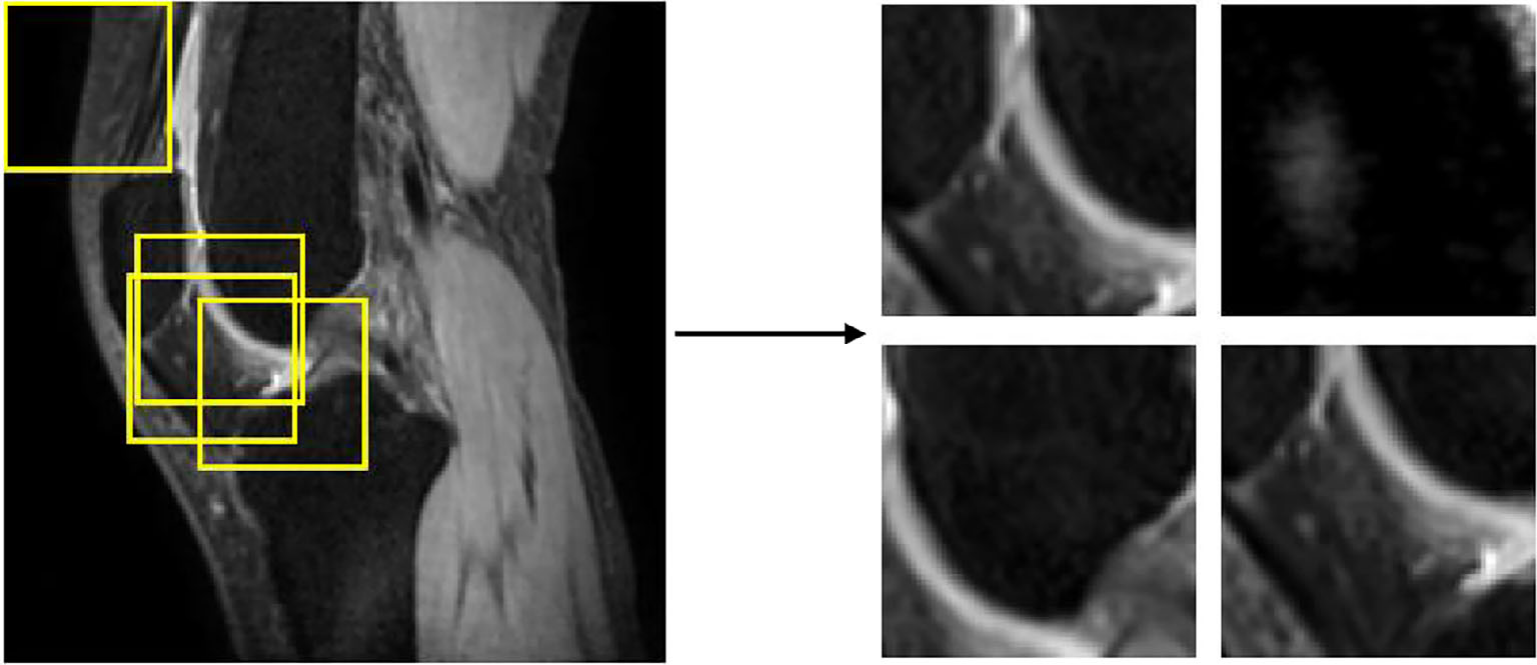
An example slice illustrating the random patching technique employed in 2D U‐Net. The yellow boxes indicate the patch positions. This slice was taken from $T_{1\rho }$‐weighted image (TSL = 0 ms) of a healthy volunteer (22 years female, BMI = 20.76 kg/m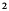). ROI = region of interest, TSL = spin lock time, and BMI = body mass index.

The 2D U‐Net model was fed with patched data with dimensions set to 64 × 64 pixels. Illustrated in Figure 3, patching enhanced the visibility of the cartilage ROI, which is relatively small compared to the entire slice. During training, the patches were randomly cropped from 2D slices, with a higher probability of selecting regions around the cartilage ROI. Augmentations were applied to the patches, including random flip, rotation, translation, and Gaussian noise addition, to enhance data diversity and prevent overfitting. When testing, we employed a sliding window strategy (window size 64 × 64 pixels) to feed every pixel from the slices containing a cartilage ROI into the 2D U‐Net model. We further compiled the output slices of $T_{1\rho }$ predictions to form 3D volumes based on their spatial positions for unified volume‐based statistical analysis.

##### 1D MLP architecture

**FIGURE 4:**
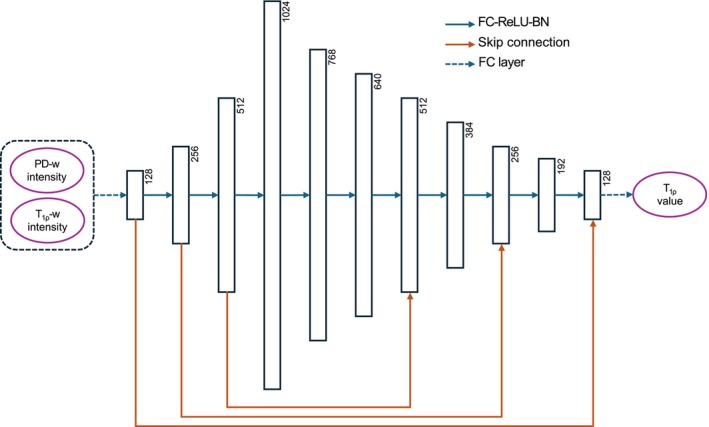
Architecture of the 1D MLP model. The PD‐weighted and $T_{1\rho }$‐weighted images were concatenated and input to the model in two channels. Legend *FC‐ReLU‐BN* means three layers in this order. MLP = multi layer perceptron, PD‐w = proton density‐weighted, $T_{1\rho }$‐w = $T_{1\rho }$‐weighted, FC = fully connected, and BN = batch normalization.

As illustrated in Figure 4, the MLP architecture was adapted from Zhang et al.^27^ This model accepts voxel intensities as the input and outputs the corresponding $T_{1\rho }$ values. We extracted voxel intensities from the cartilage ROI in the preprocessed images and ground truth $T_{1\rho }$ to form 1D vectors. These 1D vectors were subsequently fed into the MLP model for training. During evaluation, the 1D $T_{1\rho }$ prediction vectors from the MLP model were reconstructed back into 3D volumes based on the voxel positions. This extraction process ensured a unified, volume‐based statistical analysis across all methods and experiments.

The MLP model was trained for 1 000 epochs with a batch size of 512. It was optimized using L1 loss and the RMSProp optimizer^28^ (initial learning rate = 0.001, weight decay = 0.0003), with exponential learning rate decay at a rate of 0.9.

### 2.4 Experiment design

We designed our experiments to address three key questions below.

- How do the selection between PD‐weighted and baseline $T_{1\rho }$‐weighted (TSL = 0) images, and the choice of TSL in $T_{1\rho }$ imaging, affect the precision of $T_{1\rho }$ estimation using deep learning?
- What is the effect of employing different deep learning models on $T_{1\rho }$ mapping?
- Does zeroing out non‐ROI pixels in input images improve the 2D U‐Net's $T_{1\rho }$ quantification compared to unmasked data?

#### 2.4.1 Experiment 1: Input data

In this experiment, we investigated $T_{1\rho }$ quantification across six combinations of $I_{0}$ and $I_{k}$ in Equation (1). These combinations are detailed in Table 3. We selected the best‐performing deep learning models for each combination and compared their statistical metrics.

**TABLE 3:** $I_{0}$ and $I_{k}$ combinations.

No.	$I_{0}$	$I_{k}$	TSL 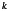
1	PD‐w^a^, ^b^	$T_{1\rho }$‐w^c^	10 ms
2	PD‐w^b^	$T_{1\rho }$‐w	30 ms
3	PD‐w^b^	$T_{1\rho }$‐w	50 ms
4	$T_{1\rho }$‐w TSL = 0 ms	$T_{1\rho }$‐w	10 ms
5	$T_{1\rho }$‐w TSL = 0 ms	$T_{1\rho }$‐w	30 ms
6	$T_{1\rho }$‐w TSL = 0 ms	$T_{1\rho }$‐w	50 ms

^a^ PD‐weighted FSE image.

^b^ Non‐linear least squares (NLLS) fitting is not applicable to this combination, as it does not conform to Equation (1).

^c^ $T_{1\rho }$‐weighted image.

#### 2.4.2 Experiment 2: Deep learning model

Deep learning‐based neural networks, such as U‐Net and MLP, have been utilized in literature for various compositional MRI prediction tasks. The 2D U‐Net and 1D MLP models mentioned above were used to investigate their performance in our specific context. Both models were trained and tested on the previously mentioned six combinations of $I_{0}$ and $I_{k}$, and we aimed to identify the most effective model under each scenario.

#### 2.4.3 Experiment 3: ROI

**FIGURE 5:**
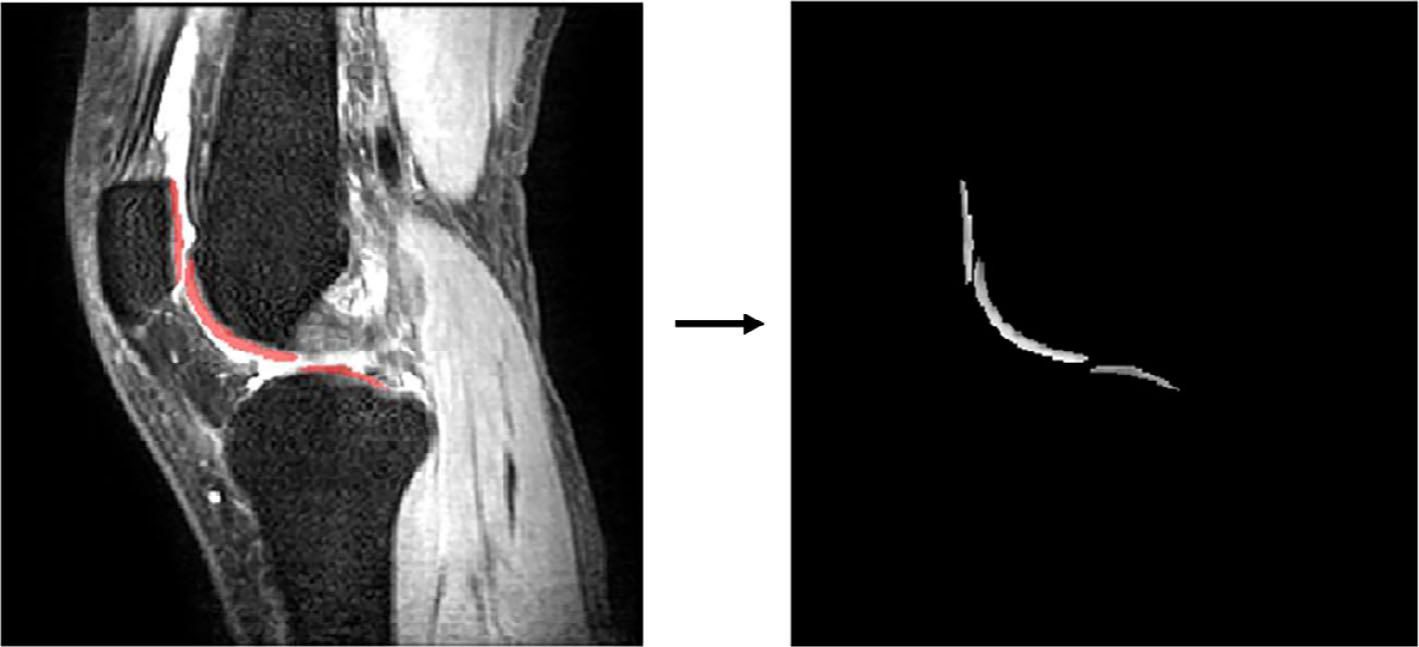
An illustration of masking ROI. The red overlay depicts the region extracted from this slice. This slice was obtained from the $T_{1\rho }$‐weighted image (TSL = 0 ms) of a healthy volunteer (22 years female, BMI = 20.76 kg/m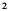). ROI = region of interest, TSL = spin lock time, and BMI = body mass index.

The $T_{1\rho }$‐weighted images on the knee have regions that do not conform to Equation (1), such as areas of bone marrow. This raises the question of whether employing ROIs in the input PD‐weighted and $T_{1\rho }$‐weighted images to constrain the 2D U‐Net could enhance the prediction performance. To investigate this, we introduced the ROI masks to zero out the voxels outside the ROI for the input images. An example of this masking operation is illustrated in Figure 5. In this context, the loss and gradient of the 2D U‐Net would be computed using only the voxel intensities within the ROI, while other voxels did not participate in the optimization process. Similarly, this experiment was conducted across all six combinations of $I_{0}$ and $I_{k}$. We compared the prediction performance of the masked and unmasked 2D U‐Net across all $I_{0}$–$I_{k}$ combinations.

#### 2.4.4 Ground truth and reference

Our ground truth was derived from a $T_{1\rho }$ quantification fitted using a conventional NLLS method with four $T_{1\rho }$‐weighted images (TSL = 0, 10, 30, 50 ms). The NLLS fitting algorithm is described in Supporting Information S2. All metrics and experiments were evaluated against this ground truth. The authors, including a musculoskeletal radiologist (F. Xiao) with 10 years of experience, manually segmented the cartilage regions to create the cartilage ROI for various training and evaluation processes.

We performed two‐point NLLS fitting for all possible combinations of $I_{0}$ and $I_{k}$ using the signal equation (Equation 1), thereby establishing a benchmark for two‐point $T_{1\rho }$ predictions using deep learning relative to the ground truth. It should be noted that NLLS fitting yields inaccurate $T_{1\rho }$ values for the three combinations involving PD‐weighted images, as these do not conform to Equation (1), and the corresponding “signal equation” for these combinations remains undetermined.

#### 2.4.5 Training and evaluation

Given the limited sample size, all training and validation procedures were performed using five‐fold cross‐validation to enhance robustness, without employing a separate test set. The same five‐fold partitioning was consistently applied across all experiments to ensure a uniform evaluation protocol. This five‐fold cross‐validation approach enabled a comprehensive assessment of the proposed methods on all 40 data samples. The cross‐validation procedure was executed as follows:

1. The 40 samples were manually partitioned into five mutually exclusive splits, with careful consideration to ensure that gender and OA severity were approximately balanced across the splits.
2. Five models were trained using identical hyperparameters, each utilizing four of the five splits for training and the remaining split for prediction.
3. Predictions from the five models were aggregated to yield a complete set of predictions for all 40 samples, upon which performance metrics were computed.

We employed five evaluation metrics to statistically analyze the $T_{1\rho }$ prediction performance of the aforementioned three experiments. We trained and evaluated all deep learning models and experiments using cross‐validation with the same five‐fold split, while the ground truth and reference NLLS fitting were directly conducted on all subjects. The metrics were calculated at the subject level, and we reported the means and standard deviations of each metric across the 40 subjects in our dataset.

We categorized the first four metrics into two types: voxel‐wise and regional errors. Voxel‐wise errors assess the absolute errors, while the regional errors are more aligned to the application of compositional MRI techniques such as $T_{1\rho }$, where the regional average of the quantification is typically involved.

The voxel‐wise errors were assessed using two common metrics: MAE and mean absolute percentage errors (MAPE). The equations for calculating MAE and MAPE for a single sample are presented in the Equations (3) and (4), where n represents the number of voxels within the ROI, ${ŷ}_{i}$ denotes predicted, and $y_{i}$ corresponds to the ground truth $T_{1\rho }$ values of the voxel.

(3) $$\text{MAE}=\frac{1}{n}\overset{n}{\underset{i=1}{\sum }}\left|y_{i}-{ŷ}_{i}\right|$$

(4) $$\text{MAPE}=\frac{1}{n}\overset{n}{\underset{i=1}{\sum }}\frac{\left|y_{i}-{ŷ}_{i}\right|}{\left|y_{i}\right|}$$

The regional errors were also assessed using two metrics: regional error (RE) and regional percentage error (RPE). The calculation for these metrics for one sample are presented as Equations (5) and (6), where n represents the number of voxels within the ROI, ${ŷ}_{i}$ denotes predicted, and $y_{i}$ corresponds the ground truth $T_{1\rho }$ values of the point.

(5) $$\text{RE}=\left|\frac{1}{n}\overset{n}{\underset{i=1}{\sum }}y_{i}-\frac{1}{n}\overset{n}{\underset{i=1}{\sum }}{ŷ}_{i}\right|$$

(6) $$\text{RPE}=\frac{\left|\frac{1}{n}\sum_{i=1}^{n}y_{i}-\frac{1}{n}\sum_{i=1}^{n}{ŷ}_{i}\right|}{\left|\frac{1}{n}\sum_{i=1}^{n}y_{i}\right|}$$

Additionally, we introduced a metric to quantify the bias associated with each method and each $I_{0}$–$I_{k}$ combination. The bias metric is defined as the difference between the means of the predicted and ground truth $T_{1\rho }$ values, as well as the percentage difference relative to the mean ground truth value

(7) $$\text{Bias}=\frac{1}{n}\overset{n}{\underset{i=1}{\sum }}{ŷ}_{i}-\frac{1}{n}\overset{n}{\underset{i=1}{\sum }}y_{i}$$

(8) $$\text{\% Bias}=\frac{\text{Bias}}{\frac{1}{n}\sum_{i=1}^{n}y_{i}}$$

When assessing performance, we established a target RPE of less than 5% for our method across all experiments.

### 2.5 Implementation

The preprocessing methods were implemented using various Python^29^ packages, including ANTsPy^23^ and Scikit‐Image,^30^ which were utilized for image registration and preprocessing. For constructing the deep learning‐based neural network, we leveraged PyTorch,^31^ PyTorch Lightning,^32^ and MONAI.^33^ Additionally, ITK‐SNAP^34^ was employed to prepare ROI labels, while MATLAB (The MathWorks, Inc., Natick, Massachusetts, USA) was utilized for executing NLLS $T_{1\rho }$ fitting.

## 3 RESULTS

We presented our comprehensive result in Table 4. For the sake of clarity and organization, we have categorized our interpretations according to the experimental framework. Our analysis indicates that across all $I_{0}$–$I_{k}$ combinations, the RPE of the best‐performing deep learning models consistently remained below our predefined threshold of 5%, and only minimal bias was found on these best‐performing models.

**TABLE 4:** Experiment results.

Model	Metrics^a^	PD‐w^b^ TSL^c^ = 10 ms	PD‐w TSL = 30 ms	PD‐w TSL = 50 ms	TSL = 0 ms TSL = 10 ms	TSL = 0 ms TSL = 30 ms	TSL = 0 ms TSL = 50 ms
2D U‐Net unmasked	Bias^d^ (ms)	−**0.37**	−**0.34**	**0.95**	**0.55**	1.19	1.10
	Bias^d^ (%)	−**0.82**	−**0.76**	**2.10**	**1.21**	2.63	2.43
	MAE (ms)	10.29 ± 2.48	9.09 ± 1.92	8.48 ± 1.56	8.92 ± 2.19	6.62 ± 1.18	6.54 ± 1.05
	MAPE (%)	25.39 ± 5.50	22.31 ± 4.48	21.75 ± 4.65	21.93 ± 4.85	17.08 ± 3.51	17.75 ± 3.99
	RE (ms)	**1.85** ± **1.29**	**1.74** ± **0.89**	**1.67** ± **1.36**	**1.35** ± **1.07**	1.23 ± 0.84	1.23 ± 0.92
	RPE^d^ (%)	**4.09** ± **2.66**	**3.86** ± **1.97**	**3.81** ± **3.21**	**2.98** ± **2.35**	2.66 ± 1.64	2.72 ± 1.97
2D U‐Net masked	Bias (ms)	−3.38	−3.21	−1.95	−2.95	−**0.66**	−0.04
	Bias (%)	−7.46	−7.08	−4.30	−6.51	−**1.45**	−0.09
	MAE (ms)	**9.94** ± **3.15**	8.32 ± 2.53	7.34 ± 2.18	8.49 ± 3.08	4.96 ± 1.75	4.71 ± 1.72
	MAPE (%)	25.74 ± 21.20	21.38 ± 18.03	20.38 ± 19.33	21.66 ± 17.90	15.23 ± 19.30	15.82 ± 19.42
	RE (ms)	3.91 ± 2.86	3.67 ± 2.70	2.96 ± 2.23	2.89 ± 2.70	0.92 ± 1.49	0.77 ± 1.60
	RPE (%)	8.53 ± 5.61	8.05 ± 5.47	6.65 ± 5.06	6.23 ± 5.19	2.28 ± 4.49	2.00 ± 4.88
1D MLP^e^	Bias (ms)	−3.96	−3.09	−1.92	−2.66	−0.69	−**0.02**
	Bias (%)	−8.74	−6.82	−4.24	−5.86	−1.52	−**0.04**
	MAE (ms)	10.52 ± 3.66	**8.19** ± **2.94**	**6.78** ± **2.49**	**8.78** ± **3.01**	**3.40** ± **1.20**	**2.74** ± **0.82**
	MAPE (%)	**22.43** ± **5.06**	**16.74** ± **3.92**	**14.78** ± **4.12**	**18.23** ± **4.52**	**7.48** ± **2.17**	**7.91** ± **2.74**
	RE (ms)	5.12 ± 4.36	4.39 ± 3.61	3.22 ± 2.86	3.43 ± 2.95	0.78 ± 0.62	**0.49** ± **0.35**
	RPE (%)	10.82 ± 7.62	9.41 ± 6.77	6.96 ± 5.61	7.22 ± 5.12	1.69 ± 1.22	**1.10** ± **0.83**
NLLS^f^	Bias (ms)	45.47	33.95	24.65	3.84	−0.59	0.48
	Bias (%)	100.25	74.85	54.36	8.46	−1.31	1.05
	MAE (ms)	49.40 ± 5.72	36.72 ± 7.45	26.81 ± 6.24	13.98 ± 4.40	3.55 ± 1.27	2.83 ± 0.83
	MAPE (%)	60.61 ± 10.86	45.42 ± 4.42	36.02 ± 4.65	30.39 ± 13.73	8.32 ± 2.59	7.47 ± 2.07
	RE (ms)	45.61 ± 10.58	34.14 ± 10.29	24.83 ± 8.22	4.16 ± 2.87	**0.71** ± **0.49**	0.66 ± 0.47
	RPE (%)	49.45 ± 9.25	42.03 ± 10.14	34.64 ± 9.37	8.32 ± 5.48	**1.62** ± **1.11**	1.43 ± 1.04

*Note*: Metric results (except bias) were shown as mean ± standard deviation among the samples. The **bold** text shows the best model in a given metric and $I_{0}$–$I_{k}$ combinations. The header row of the last six columns shows the $I_{0}$–$I_{k}$ combinations, $I_{0}$ at the top and $I_{k}$ at the bottom line. TSL = *x* ms represents a $T_{1\rho }$‐weighted image prepared by the given *x* ms of TSL.

^a^ MAE = mean absolute error, MAPE = mean absolute percentage error, RE = regional error, RPE = regional percentage error.

^b^ PD‐w = proton density (PD)‐weighted FSE MRI.

^c^ TSL = spin lock time.

^d^ Metrics were calculated with the mean of the ground truth $T_{1\rho }$, 45.36ms.

^e^ MLP = multi‐layer perceptron.

^f^ NLLS = non‐linear least square.

The results presented in this section were computed across the entire cartilage region for each subject. Additionally, we provided the corresponding results for four distinct cartilage subregions—femoral, lateral tibial, medial tibial, and patellar—in Supporting Information S3.

### 3.1 Experiment 1: Input data

Columns 3, 4, and 5 of Table 4 present the $T_{1\rho }$ prediction performance of three deep learning models for three $I_{0}$–$I_{k}$ combinations involving PD‐weighted images (Nos. 1, 2, and 3 in Table 3). We observed that the optimal bias, RE, and RPE values exhibited minimal differences from the ground truth $T_{1\rho }$ values fitted by NLLS using four $T_{1\rho }$‐weighted images. Furthermore, we found that as the TSL increased, all metrics decreased across all models, except for the bias metric of the 2D U‐Net (unmasked), which did not follow this trend.

Meanwhile, two‐point $T_{1\rho }$ predictions (Nos. 4, 5, and 6 in Table 3) are shown in columns 6, 7, and 8 of Table 4. All three deep learning models outperformed NLLS fitting when only two $T_{1\rho }$‐weighted images were used. Compared to the PD‐weighted cases, these two‐point $T_{1\rho }$ predictions demonstrated superior performance and exhibited the same trend of improved prediction performance with increasing TSL.

**FIGURE 6:**
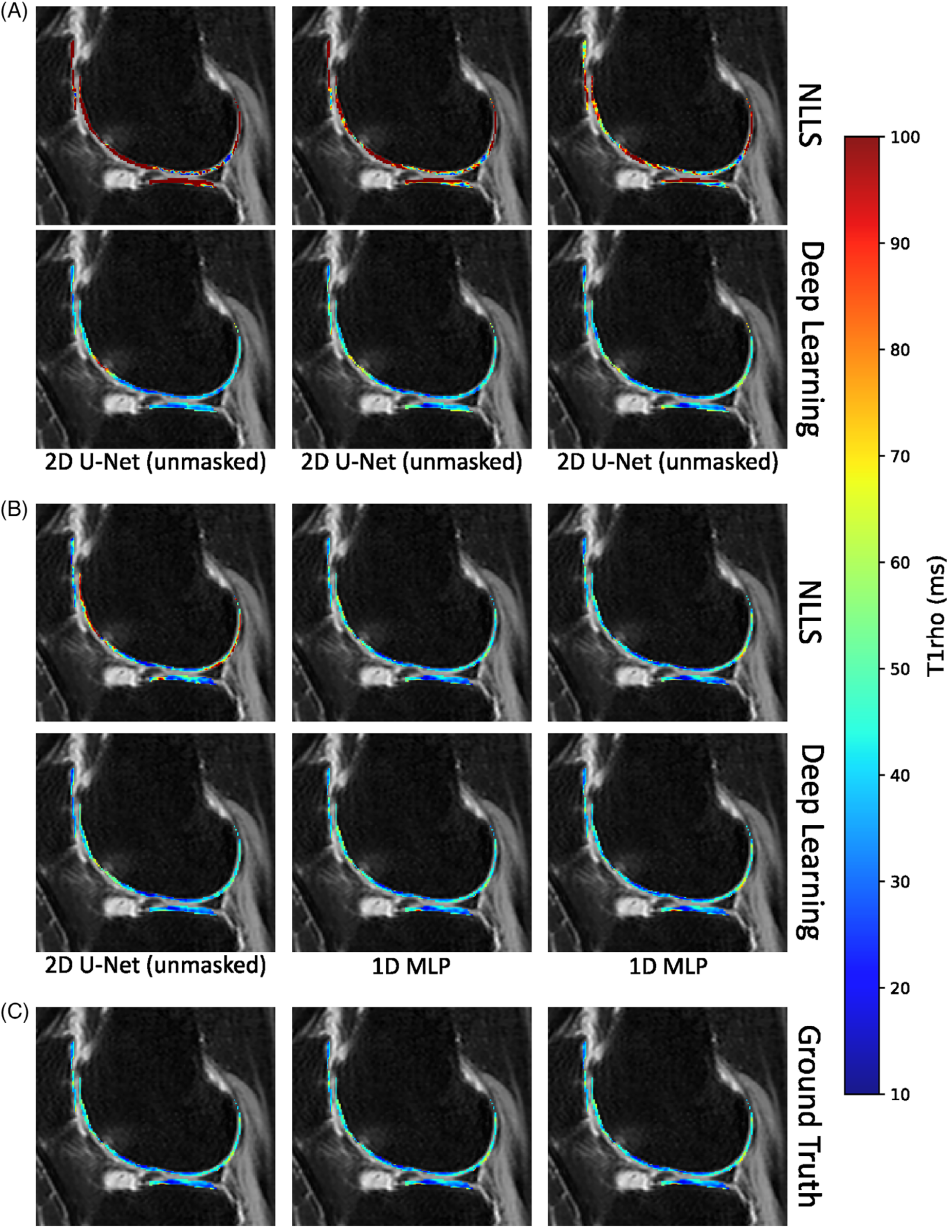
A representative slice from a patient with moderate OA (57‐year‐old female, BMI = 29.10 kg/m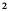, right knee). The figure illustrates $T_{1\rho }$ maps obtained via NLLS fitting, deep learning prediction, and ground truth reference. (A) shows $T_{1\rho }$ maps where $I_{0}$ is a PD‐weighted image; (B) displays maps where $I_{0}$ is a $T_{1\rho }$‐weighted image; and (C) presents the ground truth. The columns correspond to three distinct $I_{k}$ configurations, with $T_{1\rho }$‐weighted images (TSL = 10 ms/30 ms/50 ms) arranged from left to right. Model names are indicated directly below the deep learning predictions. The deep learning results are derived from the best‐performing model for each $I_{0}$–$I_{k}$ combination. The ground truth maps are repeated three times to facilitate interpretation. NLLS = non‐linear least squares fitting, OA = osteoarthritis, BMI = body mass index, PD = proton density, and TSL = time of spin‐lock.

A visualization of $T_{1\rho }$ maps fitted and predicted by NLLS and deep learning models is presented in Figure 6. Additional visualizations are provided in Supporting Information S4.

### 3.2 Experiment 2: Deep learning model

For combinations involving PD‐weighted images, we observed that the 2D U‐Net (unmasked) exhibited the lowest bias, RE, and RPE, whereas the 1D MLP achieved the lowest MAE and MAPE, except for the combination of PD‐weighted and $T_{1\rho }$‐weighted images at TSL = 10 ms. Similarly, in two‐point $T_{1\rho }$ prediction, a comparable pattern was observed when utilizing $T_{1\rho }$‐weighted images at TSLs of 0 and 10 ms.

Notably, the 2D U‐Net (unmasked) appeared more adept at predicting and harmonizing $T_{1\rho }$ values for $I_{0}$–$I_{k}$ combinations involving PD‐weighted and $T_{1\rho }$‐weighted (TSL = 10 ms) images, as evidenced by its low RE and RPE. However, the optimal MAE and MAPE consistently corresponded to the 1D MLP model, which was not aligned with the RE and RPE results. Furthermore, the 2D U‐Net (unmasked) did not maintain its superiority in the two‐point $T_{1\rho }$ prediction task. Instead, the 1D MLP outperformed the other models when $T_{1\rho }$‐weighted images with TSLs of 10 ms and 30 ms were used.

### 3.3 Experiment 3: ROI

In this experiment, we compare the $T_{1\rho }$ prediction metrics between unmasked and ROI‐masked 2D U‐Net models. We observed that applying ROI masking generally degraded prediction performance in most scenarios, as measured by the RPE. However, the ROI‐masked 2D U‐Net outperformed its unmasked counterpart when $I_{0}$ was a $T_{1\rho }$‐weighted image (TSL = 0) and $I_{k}$ were $T_{1\rho }$‐weighted images (TSL = 30 ms and 50 ms). Notably, these two combinations coincide with those in which the 1D MLP also demonstrated superior performance. These results are summarized in the second and third rows of Table 4.

## 4 DISCUSSION

Our study used deep learning techniques to investigate the potential of utilizing PD‐weighted anatomical FSE images for $T_{1\rho }$ quantification. The proposed method differs from standard $T_{1\rho }$ fitting approaches, which calculate $T_{1\rho }$ values using an explicit mono‐exponential model. Instead, our approach predicts the $T_{1\rho }$ map directly from a PD‐weighted anatomical image and a $T_{1\rho }$‐weighted image, using a neural network trained under the supervision of a reference $T_{1\rho }$ map. Unlike conventional methods, this approach does not depend on explicit signal models between the two images, as applying mono‐exponential fitting to these images has been shown to produce erroneous results. Furthermore, this method does not involve predicting images or their corresponding k‐space data. The images are acquired using standard imaging protocols and subsequently processed by the neural network to predict the $T_{1\rho }$ map. The reliability of the prediction is assessed using a specified metric, rather than the goodness‐of‐fit metric typically used in conventional fitting methods.

To evaluate this approach, we conducted various experiments on a dataset comprising OA patients and healthy volunteers, examining the performance of deep learning prediction methods under different input data, deep learning model architectures, and preprocessing settings. Our results demonstrated that the proposed deep learning methods achieved the best RPE of less than 5% across all six $I_{0}$ and $I_{k}$ combinations, thereby substantiating our hypothesis and establishing a reliable $T_{1\rho }$ prediction method. Our experiments also demonstrated that deep learning‐based $T_{1\rho }$ prediction methods can effectively quantify $T_{1\rho }$ using only two $T_{1\rho }$‐weighted images obtained from the knee. Similar findings have been reported in $T_{1\rho }$ imaging studies of the liver.^8^, ^35^

In this study, we investigated the selection of TSL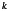 (in the $T_{1\rho }$ signal Equation 1) using the corresponding $I_{k}$ images. In spin‐lock‐based acquisitions, the maximum TSL is limited by the RF amplifier configurations and SAR.^36^ Meanwhile, as reported by Zibetti et al., the optimal TSL for a given tissue closely approximates its intrinsic $T_{1\rho }$ value.^37^ In our study, the optimal TSL was determined to be 50 ms, corresponding to a mean $T_{1\rho }$ of 45.36 ms. Notably, the proposed deep learning approach was able to predict $T_{1\rho }$ with minimal errors (relative percentage error less than 5%) even when utilizing non‐optimal TSLs. This capability facilitates the acquisition of $T_{1\rho }$ maps using shorter TSLs, thereby reducing both the SAR and the demands placed on RF hardware.

Our experiments were conducted to elucidate the efficacy of deep learning methods under various data inputs and model configurations. We aimed to identify which model performed optimally with a given data input. In our proposed scenario of $T_{1\rho }$ prediction, the deep learning models, namely neural networks, operated as universal approximators^9^ of the $T_{1\rho }$ signal equation (Equation 1). It was anticipated that these neural networks would effectively predict $T_{1\rho }$ from only one to two $T_{1\rho }$‐weighted images, exhibiting comparable performance to the standard NLLS fitting using multiple $T_{1\rho }$‐weighted images. Nonetheless, mathematically, we were tasked with predicting $T_{1\rho }$ from the minimum number of images, where the algorithms must balance noise and out‐of‐distribution (OOD) signals to achieve accurate prediction.

Our comparisons with two classic deep learning models, the 2D U‐Net and the 1D MLP, revealed that the 2D U‐Net was more adept at addressing the challenges posed by noise and OOD data. This assertion is substantiated by the trend analysis of metrics against the $I_{0}$–$I_{k}$ combinations: The 2D U‐Net (unmasked) outperformed the 1D MLP in all combinations involving PD‐weighted images and $T_{1\rho }$‐weighted images collected at a TSL of 10 ms. Conversely, the 1D MLP surpassed the performance of the 2D U‐Net (masked and unmasked) in the remaining combinations. We propose that this distinction arose due to the 2D U‐Net's capacity to capture spatial information from the input images,^38^ which 1D MLP lacked. Moreover, the convolution operation within the 2D U‐Net facilitated the removal of noise signals.^39^ These attributes endowed the 2D U‐Net with a robust ability to leverage additional information and balance the OOD data for accurate $T_{1\rho }$ quantification. Conversely, the 1D MLP approximated the $T_{1\rho }$ signal equation more effectively in scenarios characterized by minimal noise and OOD signals.

In accordance with the universal approximation theorem^9^ and the assumptions underlying $T_{1\rho }$ imaging, we conducted experiments in which regions outside the cartilage ROI were masked for the 2D U‐Net. We posited that the 2D U‐Net would more effectively focus on regions adhering to the $T_{1\rho }$ signal equation (Equation 1). This hypothesis is valid only when the input data strictly conforms to the $T_{1\rho }$ signal equation, as demonstrated in two scenarios: When $I_{0}$ consisted of $T_{1\rho }$‐weighted images (TSL = 0) and $I_{k}$ comprised $T_{1\rho }$‐weighted images (TSL = 30 and 50 ms). In contrast, the unmasked 2D U‐Net generally outperformed its masked counterpart in other scenarios. Given that CNN‐based models such as U‐Net^24^ are designed to capture and encode spatial information from training data,^38^, ^39^ we interpret these findings as evidence of the importance of voxels outside the ROI in enabling the 2D U‐Net to achieve accurate $T_{1\rho }$ quantification within the ROI, particularly for OOD data. Although the intensities of these voxels neither conform to the signal equation (Equation 1) nor directly contribute to the $T_{1\rho }$ prediction task, they nonetheless provide critical anatomical context and spatial features pertinent to knee image analysis.

$T_{1\rho }$ imaging holds great potential as a diagnostic tool for cartilage assessment; however, its limited adoption in clinical practice highlights the need for strategies to integrate it into routine workflows. One promising approach is to derive $T_{1\rho }$ maps directly from conventional anatomical images, such as clinical FSE sequences, which are commonly used for knee imaging. This method could address the challenge of additional scan time required for $T_{1\rho }$ MRI by incorporating $T_{1\rho }$ quantification into standard clinical protocols. Previous studies support this strategy: Santyr et al. demonstrated that Carr‐Purcell‐Meiboom‐Gill (CPMG) acquisitions can replicate spin‐locking behavior using density matrix theory when the echo spacing matches the spin‐lock frequency,^40^ and Gold et al. demonstrated this in in vivo experiments.^41^ The ultimate goal is to derive $T_{1\rho }$ values entirely from standard clinical protocols without requiring additional acquisitions at non‐zero TSL, enabling simultaneous anatomical and biochemical assessment of knee cartilage within routine imaging workflows.

Despite the potential benefits of our proposed approach, several limitations must be acknowledged. First, the study relied on a retrospectively acquired and relatively small dataset. Future research should incorporate test‐retest experiments and validate the method in larger patient populations to establish its clinical relevance. Second, the $T_{1\rho }$ quantification protocol employed standard knee imaging parameters typically used in $T_{1\rho }$‐weighted imaging, which have lower resolution and a different field of view compared to the PD‐weighted imaging. Although registration was implemented to address this issue, the relatively large standard deviation indicates that the acquisition mismatch may have negatively impacted the results. Third, while three‐dimensional GRE pulse sequences^14^, ^17^ are commonly used for $T_{1\rho }$ mapping, our investigated method is based on 3D FSE acquisition. Both approaches utilize variable flip angle schemes, but 3D FSE's typical flip angle variations result in rapid signal fluctuations during initial echoes.^19^ Consequently, these initial echoes are not acquired to avoid artifacts, leading to relatively long echo times compared to GRE acquisitions. Further research is needed to explore alternative pulse sequence designs for $T_{1\rho }$ prediction in our proposed approach. Finally, the pulse sequence used for $T_{1\rho }$‐weighted acquisition followed a conventional design for quantitative $T_{1\rho }$ imaging, requiring magnetization reset at the beginning of each TR to ensure consistent magnetization for the spin‐lock preparation. While this consistency enables the use of a simple mono‐exponential model in conventional $T_{1\rho }$ fitting methods, our proposed approach predicts the $T_{1\rho }$ map from a $T_{1\rho }$‐prepared image and a PD‐weighted image without relying on such fitting models. This method potentially offers greater flexibility regarding magnetization reset requirements, though additional studies are needed to fully investigate this aspect of pulse sequence design for $T_{1\rho }$ imaging.

In summary, we have proposed accelerated acquisition and processing methods for knee $T_{1\rho }$ quantification using PD‐weighted and $T_{1\rho }$‐weighted FSE MRI alongside deep learning. We demonstrated our approach on a dataset collected from OA patients and healthy volunteers. Notably, our approach exhibits compatibility with $T_{1\rho }$‐weighted images acquired with a shorter TSL of 10ms, thereby enabling the reduction of RF power and SAR during optimized $T_{1\rho }$ acquisition. To further refine our method, we conducted experiments to explore the effects of various design choices, providing valuable insights into selecting optimal deep learning models for predicting $T_{1\rho }$ in clinical MRI. The proposed approach has the potential to facilitate the incorporation of advanced quantitative MRI methods into routine clinical practice, ultimately benefiting patients and the broader population.

## CONFLICT OF INTEREST STATEMENT

Queenie Chan is an employee at Philips Healthcare. Other authors declare no potential conflict of interest.

## Supporting information


**Data S1.** Supporting Information.
