# Supplementary material for: Utilizing 3D fast spin echo anatomical imaging to reduce the number of contrast preparations in T1ρ quantification of knee cartilage using learning‐based methods
Source: Magn Reson Med. 2025 Aug 5;94(6):2745–57. doi: 10.1002/mrm.70022 (PMC12501725; doi:10.1002/mrm.70022)
Supplement: Supplementary file 1 — Data S1. Supporting Information. [file MRM-94-2745-s001.pdf]

Utilizing 3D Fast Spin Echo Anatomical Imaging to Reduce the Number of Contrast Preparations in $T_{1\rho}$ Quantification of Knee Cartilage Using Learning-Based Methods

Junru Zhong, Chaoxing Huang, Ziqiang Yu, Fan Xiao, Thierry Blu, Siyue Li, Queenie Chan, Tim-Yun Michael Ong, Ki-Wai Kevin Ho, James F. Griffith, Weitian Chen

Supplementary Materials

S1 Registration

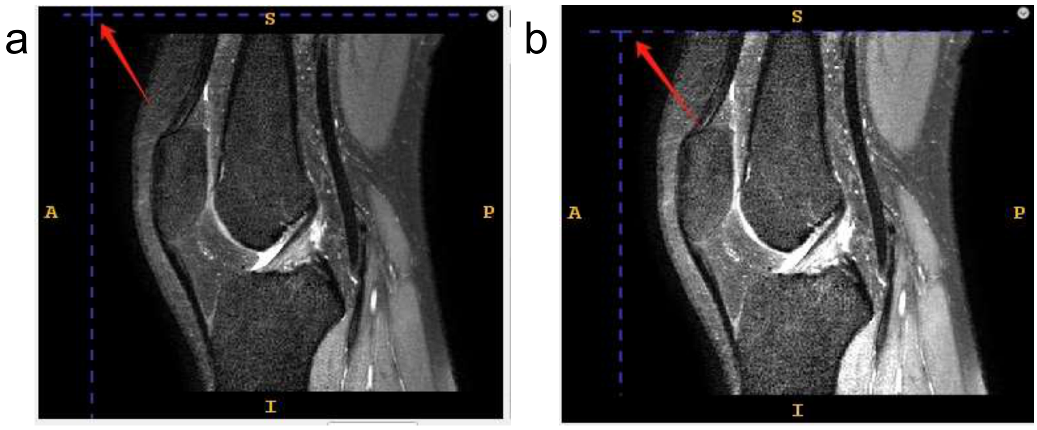

Figure S1: Screenshots in the ITK-SNAP[1] viewer that demonstrate the FOV crop (**a**, before FOV crop; **b**, after FOV crop). The red arrows point to the top-left edges of the slices. The blue lines are the crosshair. The crosshair position in **a** shows the blank fill. Note, FOV = field of view.

S1.1 Procedure

The registration between proton density (PD)-weighted and $T_{1\rho}$-weighted images was conducted through the following steps.

1. **Field of view (FOV) alignment**. The PD-weighted images were acquired using a rectangular FOV, whereas the $T_{1\rho}$-weighted images were obtained with a square FOV in the sagittal plane. It was observed that the sagittal slices of the images contained blank-filled regions at either end. To align the centers of the FOVs between the two image types, the blank areas were cropped

by precisely calculating the spacing and image dimensions. An example of this FOV cropping is illustrated in Figure S1. This adjustment reduced the image size from 292 × 512 × 512 voxels to 292 × 512 × 443 voxels.

2. **Image size alignment**. Following the FOV cropping, the PD-weighted images were resized to 292 × 512 × 443 voxels. Since our $T_{1\rho}$-weighted images had a smaller image size of 44 × 256 × 256 voxels, the PD-weighted images were resized using ANTsPy[2] to match the dimension of the $T_{1\rho}$-weighted images.

3. **Three-step registration**. A three-step registration process was applied to the PD-weighted images, with the $T_{1\rho}$-weighted images serving as the fixed reference. The registration involved sequential application of rigid, affine, and symmetric deformable transformations[2]. Each transformation utilized the output of the preceding step, and the final symmetric deformable transformation produced the images used for $T_{1\rho}$ fitting.

S1.2 Evaluation

The evaluation of the registration was conducted using the Structural Similarity Index (SSIM). As suggested by Rohlfing, regional similarity metrics may provide a more accurate reflection of actual registration performance[3]. Accordingly, we report the mean ± standard deviation of the SSIM values for 40 image pairs under three distinct conditions:

- 0.73±0.04 for the full image areas.
- 0.66±0.04 for images cropped to the bounding box of the cartilage region of interest (ROI).
- 0.97±0.01 for images cropped to the bounding box of the cartilage ROI and further masked by the cartilage ROI.

It is important to note that SSIM can be influenced by voxel intensity, which may explain the observed variations among the three SSIM values. The voxel intensity in the regions used for SSIM calculation was significantly affected by two factors: acquisition protocols and fat suppression. The moving and fixed images in this registration task were acquired using two markedly different protocols, resulting in substantial differences in voxel intensities and contrasts. Additionally, a considerable portion of the images consisted of bone marrow, where fat suppression was applied. This suppression introduced noise into the voxel intensities within the region.

Given these limitations and Rohlfing's recommendation[3], we determined that the SSIM calculated on the cropped and masked images most accurately reflects our specific use case. Consequently, we interpret the SSIM value of 0.97±0.01 as indicative of satisfactory registration performance.

S2 NLLS Fitting

Task setting We set the signal equation in this NLLS fitting task as Equation S1 below:

$$M^{tsl} = ae^{-R_{1\rho}^{tsl}} \tag{S1}$$

where M^{tsl} is the magnitude at the given TSL, $R_{1\rho} = \frac{1}{T_{1\rho}}$, and where a is some parameter. Given M^{tsl} and TSL, we want to estimate $R_{1\rho}$ and a.

Objective function We choose to quantify the fitting error between the actual measurements and the signal model by the mean squared error (MSE, Equation S2).

$$\text{MSE}(a, R_{1\rho}) = \sum_{tsl} |M^{tsl} - ae^{-R_{1\rho}tsl}|^2 \tag{S2}$$

Minimizing this objective function over a and $R_{1\rho}$ is performed by first minimizing over a. This results in:

$$a(R_{1\rho}) = \frac{f_1(R_{1\rho} - a_1(R_{1\rho})f_0(R_{1\rho})}{a_2(R_{1\rho}) - a_1^2(R_{1\rho})} \tag{S3}$$

where the functions, f_0, f_1, a_1, and a_2 are defined as follows:

$$a_1(R_{1\rho}) = \frac{1}{\#tsl} \sum_{tsl} e^{-R_{1\rho}tsl} \tag{S4}$$

$$a_2(R_{1\rho}) = \frac{1}{\#tsl} \sum_{tsl} e^{-2R_{1\rho}tsl} \tag{S5}$$

$$f_0(R_{1\rho}) = \frac{1}{\#tsl} M^{tsl} \tag{S6}$$

$$f_1(R_{1\rho}) = \frac{1}{\#tsl} \sum_{tsl} M^{tsl} e^{-R_{1\rho}tsl} \tag{S7}$$

Note that these functions can be implemented efficiently as vectorized operations in usual scientific packages (e.g., MATLAB, Python).

The objective function is now a function of $R_{1\rho}$ only:

$$J(R_{1\rho}) = \text{MSE}(a(R_{1\rho}), R_{1\rho}) \tag{S8}$$

and its minimum provides the optimal value of $R_{1\rho}$.

Optimization method Minimizing $J(R_{1\rho})$ over $R_{1\rho}$ can be achieved by a simple line search strategy (i.e., try out many values of $R_{1\rho}$ and keep the best one), but we observed that, in general, this objective function has only one minimum within the expected range of $R_{1\rho}$. Therefore, we resorted to the (faster) dichotomic strategy that is detailed below:

1. Starting from a range $[R_{\min}, R_{\max}]$, evaluate the derivative of the criterion J at the midpoint $R = (R_{\min} + R_{\max})/2$.
2. If the derivative is positive (meaning J increases at this point), we know that the minimum of J lies in the interval $[R_{\min}, R]$, hence update $R_{\max} = R$.
3. If the derivative is negative (meaning J decreases at this point), we know that the minimum of J lies in the interval $[R, R_{\max}]$, hence update $R_{\min} = R$.
4. Repeat the above three steps until $R_{\min} - R_{\max}$ is smaller than a pre-defined accuracy. Note that $R_{\min} - R_{\max}$ decreases by a factor two at each iteration.

Optimization parameters In this manuscript, we set this NLLS fitting algorithm with these parameters.

- We fit the $T_{1\rho}$ ($R_{1\rho}$) values using the magnitude image.
- $R_{min} = -0.001\text{ms}^{-1}$, and $R_{\max} = \frac{20}{\max(tsl)-\min(tsl)} = \frac{20}{50\text{ms}-0\text{ms}} = 0.4\text{ms}^{-1}$.
- Search interval, $[R_{\min}, R_{\max}]$, is $[0.001, 0.4]\text{ms}^{-1}$.
- Stopping criteria is $R_{\max} - R_{\min} < 0.001\text{ms}^{-1}$, which is reached within 9 iterations.

S3 Metric Results in Cartilage Regions

We generated the metric results from four cartilage regions, femoral, lateral tibial, medial tibial, and patellar cartilages. We reported the results in six tables, Table S1 to S6, corresponding to six I_0-I_k combinations.

From these result tables, we can interpret the followings:

- Compare to the metric results of entire cartilage regions showed in Table 4 of the main text, we observed increases on the errors in all cartilage regions.
- For each ROI, the trend of the result numbers followed the results showed in the main text, i.e., as spin-lock time (TSL) getting close to the optimal, the errors reduce.
- Comparing the results between cartilage regions, we observed the smaller cartilage regions like tibial cartilages had worse metric results.

Table S1: Cartilage region metric results. I_0: PD-weighted, I_k: $T_{1\rho}$-weighted (TSL=10ms)

ROI	Metrics	2D U-Net (unmasked)	2D U-Net (masked)	1D MLP	NLLS
Femoral Cartilage	Bias [ms (%)]	0.18 (0.38)	-3.60 (-7.74)	-3.95 (-8.49)	37.82 (81.24)
	MAE (ms)	11.28 ± 6.31	9.94 ± 3.36	10.02 ± 3.47	46.06 ± 4.80
	MAPE (%)	27.12 ± 17.59	21.29 ± 6.60	20.71 ± 5.02	120.84 ± 19.88
	RE (ms)	3.56 ± 6.72	4.33 ± 3.66	5.08 ± 4.36	37.82 ± 9.22
	RPE (%)	7.88 ± 16.31	9.13 ± 7.81	10.36 ± 7.47	82.75 ± 24.58
Lateral Tibial Cartilage	Bias [ms (%)]	3.20 (8.22)	-2.58 (-6.64)	-1.02 (-2.62)	43.60 (112.14)
	MAE (ms)	11.70 ± 9.18	9.25 ± 3.90	10.63 ± 4.50	51.00 ± 7.18
	MAPE (%)	36.36 ± 41.99	23.13 ± 8.40	27.08 ± 8.25	164.48 ± 37.84
	RE (ms)	4.79 ± 9.68	4.02 ± 2.97	5.27 ± 4.58	43.60 ± 11.93
	RPE (%)	13.45 ± 31.10	9.98 ± 6.81	12.99 ± 8.79	116.12 ± 39.99
Medial Tibial Cartilage	Bias [ms (%)]	1.96 (4.63)	-4.22 (-9.94)	-4.61 (-10.86)	42.55 (100.31)
	MAE (ms)	12.34 ± 6.67	11.25 ± 4.90	11.93 ± 5.98	49.56 ± 5.62
	MAPE (%)	35.08 ± 25.02	26.04 ± 9.49	26.93 ± 8.67	153.76 ± 37.17
	RE (ms)	4.64 ± 7.56	5.01 ± 4.58	5.95 ± 6.72	42.55 ± 10.57
	RPE (%)	11.57 ± 21.41	11.27 ± 9.00	12.83 ± 11.85	105.76 ± 39.46
Patellar Cartilage	Bias [ms (%)]	1.16 (2.35)	-6.12 (-12.39)	-7.25 (-14.66)	38.50 (77.89)
	MAE (ms)	12.84 ± 5.85	12.30 ± 6.15	13.05 ± 8.56	46.00 ± 9.17
	MAPE (%)	29.36 ± 13.95	22.99 ± 7.11	23.20 ± 8.76	122.75 ± 37.14
	RE (ms)	4.94 ± 5.27	7.06 ± 6.82	9.40 ± 9.74	38.63 ± 15.34
	RPE (%)	9.82 ± 10.70	12.97 ± 9.77	16.80 ± 13.10	84.92 ± 40.17

Metric results (except bias) were shown as mean ± standard deviation among the samples. Biases were calculated between the mean $T_{1\rho}$ values from ground truths and predictions within ROI. Note, ROI = region of interest, MAE = mean absolute error, MAPE = mean absolute percentage error, RE = regional error, RPE = regional percentage error, MLP = multi-layer perceptron, NLLS = non-linear least squares.

These observations suggest future improvements of the performance in smaller cartilage regions are demanded.

Table S2: Cartilage region metric results. I_0: PD-weighted, I_k: $T_{1\rho}$-weighted (TSL=30ms)

ROI	Metrics	2D U-Net (unmasked)	2D U-Net (masked)	1D MLP	NLLS
Femoral Cartilage	Bias [ms (%)]	0.50 (1.07)	-3.47 (-7.45)	-2.70 (-5.79)	31.78 (68.26)
	MAE (ms)	10.22 ± 6.41	8.41 ± 3.02	7.81 ± 2.64	39.27 ± 5.26
	MAPE (%)	24.66 ± 18.04	17.73 ± 5.92	15.72 ± 3.65	103.08 ± 18.76
	RE (ms)	3.30 ± 6.82	4.17 ± 3.47	4.20 ± 3.53	31.78 ± 8.11
	RPE (%)	7.37 ± 16.64	8.85 ± 7.47	8.72 ± 6.50	69.43 ± 21.16
Lateral Tibial Cartilage	Bias [ms (%)]	3.13 (8.05)	-2.30 (-5.91)	-1.65 (-4.24)	36.21 (93.13)
	MAE (ms)	10.49 ± 9.12	7.74 ± 3.11	8.41 ± 3.86	42.94 ± 7.65
	MAPE (%)	32.76 ± 41.92	19.22 ± 7.17	19.72 ± 5.91	138.04 ± 38.26
	RE (ms)	4.08 ± 9.39	3.37 ± 2.25	4.30 ± 3.93	36.25 ± 11.47
	RPE (%)	11.61 ± 30.37	8.41 ± 5.16	10.41 ± 7.37	96.99 ± 37.13
Medial Tibial Cartilage	Bias [ms (%)]	1.70 (4.01)	-3.83 (-9.04)	-4.89 (-11.52)	37.79 (89.10)
	MAE (ms)	11.20 ± 7.65	9.63 ± 3.96	9.64 ± 5.45	44.32 ± 5.87
	MAPE (%)	31.67 ± 29.12	21.92 ± 8.03	19.78 ± 6.54	138.05 ± 36.50
	RE (ms)	3.98 ± 8.64	4.67 ± 4.26	5.88 ± 6.17	37.79 ± 9.66
	RPE (%)	10.12 ± 24.62	10.47 ± 8.58	12.72 ± 10.99	94.02 ± 37.05
Patellar Cartilage	Bias [ms (%)]	0.90 (1.83)	-5.30 (-10.71)	-5.20 (-10.53)	31.00 (62.73)
	MAE (ms)	11.08 ± 5.46	10.19 ± 5.08	9.66 ± 6.87	39.44 ± 8.76
	MAPE (%)	25.21 ± 13.35	18.96 ± 6.36	16.95 ± 7.49	104.51 ± 35.29
	RE (ms)	3.87 ± 4.77	6.00 ± 5.98	7.33 ± 7.58	32.40 ± 13.75
	RPE (%)	7.67 ± 9.55	11.10 ± 9.88	13.21 ± 10.58	71.30 ± 36.04

Metric results (except bias) were shown as mean ± standard deviation among the samples. Biases were calculated between the mean $T_{1\rho}$ values from ground truths and predictions within ROI. Note, ROI = region of interest, MAE = mean absolute error, MAPE = mean absolute percentage error, RE = regional error, RPE = regional percentage error, MLP = multi-layer perceptron, NLLS = non-linear least squares.

Table S3: Cartilage region metric results. I_0: PD-weighted, I_k: $T_{1\rho}$-weighted (TSL=50ms)

ROI	Metrics	2D U-Net (unmasked)	2D U-Net (masked)	1D MLP	NLLS
Femoral Cartilage	Bias [ms (%)]	1.95 (4.20)	-2.27 (-4.86)	-1.70 (-3.65)	26.34 (56.56)
	MAE (ms)	9.58 ± 6.21	7.55 ± 2.95	6.48 ± 2.29	34.27 ± 4.67
	MAPE (%)	24.17 ± 17.71	16.77 ± 6.36	13.86 ± 4.02	90.15 ± 16.96
	RE (ms)	3.01 ± 6.67	3.71 ± 3.19	3.30 ± 2.82	26.34 ± 7.11
	RPE (%)	6.93 ± 16.25	8.04 ± 7.33	6.92 ± 5.36	57.60 ± 18.45
Lateral Tibial Cartilage	Bias [ms (%)]	3.56 (9.16)	-1.51 (-3.88)	-0.95 (-2.43)	32.40 (83.33)
	MAE (ms)	10.04 ± 9.01	6.74 ± 2.66	7.15 ± 3.14	38.32 ± 6.98
	MAPE (%)	31.89 ± 41.71	17.39 ± 6.80	17.61 ± 5.89	123.40 ± 34.25
	RE (ms)	4.08 ± 9.27	2.65 ± 1.81	3.52 ± 2.91	32.40 ± 10.40
	RPE (%)	11.60 ± 30.03	6.72 ± 4.26	8.72 ± 5.91	86.77 ± 33.40
Medial Tibial Cartilage	Bias [ms (%)]	2.56 (6.04)	-2.45 (-5.78)	-3.11 (-7.33)	34.53 (81.41)
	MAE (ms)	10.54 ± 6.86	8.61 ± 3.65	8.22 ± 4.38	41.01 ± 5.80
	MAPE (%)	30.76 ± 26.71	20.73 ± 8.55	18.24 ± 6.46	127.71 ± 33.04
	RE (ms)	3.96 ± 7.78	3.82 ± 4.10	4.71 ± 4.86	34.53 ± 9.40
	RPE (%)	10.30 ± 22.27	8.83 ± 8.76	10.40 ± 9.04	85.89 ± 34.27
Patellar Cartilage	Bias [ms (%)]	2.76 (5.59)	-3.16 (-6.39)	-2.71 (-5.49)	20.55 (41.59)
	MAE (ms)	10.25 ± 5.07	8.67 ± 4.42	7.28 ± 4.88	31.79 ± 6.95
	MAPE (%)	24.58 ± 13.36	17.19 ± 6.57	13.76 ± 6.24	82.14 ± 29.35
	RE (ms)	3.94 ± 4.53	4.46 ± 5.02	5.08 ± 5.20	23.43 ± 11.27
	RPE (%)	8.13 ± 9.43	8.48 ± 9.44	9.39 ± 7.84	51.54 ± 29.63

Metric results (except bias) were shown as mean ± standard deviation among the samples. Biases were calculated between the mean $T_{1\rho}$ values from ground truths and predictions within ROI. Note, ROI = region of interest, MAE = mean absolute error, MAPE = mean absolute percentage error, RE = regional error, RPE = regional percentage error, MLP = multi-layer perceptron, NLLS = non-linear least squares.

Table S4: Cartilage region metric results. I_0: $T_{1\rho}$-weighted (TSL=0), I_k: $T_{1\rho}$-weighted (TSL=10ms)

ROI	Metrics	2D U-Net (unmasked)	2D U-Net (masked)	1D MLP	NLLS
Femoral Cartilage	Bias [ms (%)]	1.33 (2.86)	-3.17 (-6.80)	-2.38 (-5.11)	5.03 (10.79)
	MAE (ms)	9.99 ± 6.36	8.61 ± 3.44	8.47 ± 2.76	14.46 ± 4.29
	MAPE (%)	24.13 ± 17.80	18.18 ± 6.55	17.33 ± 4.34	32.29 ± 10.23
	RE (ms)	2.64 ± 6.75	3.26 ± 3.39	3.32 ± 2.78	5.82 ± 3.92
	RPE (%)	6.00 ± 16.40	6.84 ± 7.18	6.83 ± 5.11	12.61 ± 8.73
Lateral Tibial Cartilage	Bias [ms (%)]	3.22 (8.29)	-2.37 (-6.09)	-0.70 (-1.81)	4.30 (11.06)
	MAE (ms)	10.19 ± 9.01	7.73 ± 3.57	8.25 ± 3.89	12.20 ± 6.15
	MAPE (%)	31.83 ± 41.38	18.90 ± 7.44	19.50 ± 6.85	30.93 ± 16.44
	RE (ms)	4.14 ± 9.38	2.93 ± 2.50	3.62 ± 3.92	5.86 ± 6.31
	RPE (%)	11.68 ± 30.30	7.17 ± 5.49	8.84 ± 8.56	15.01 ± 16.78
Medial Tibial Cartilage	Bias [ms (%)]	3.88 (9.15)	-3.14 (-7.41)	-2.51 (-5.91)	5.25 (12.38)
	MAE (ms)	11.59 ± 7.77	9.45 ± 4.59	9.74 ± 5.52	15.13 ± 9.21
	MAPE (%)	33.21 ± 28.88	21.42 ± 8.12	20.97 ± 8.16	35.39 ± 21.60
	RE (ms)	5.26 ± 8.63	3.55 ± 4.20	4.70 ± 5.63	8.74 ± 10.07
	RPE (%)	13.01 ± 24.30	8.00 ± 7.56	10.17 ± 9.78	19.66 ± 21.18
Patellar Cartilage	Bias [ms (%)]	1.75 (3.55)	-4.72 (-9.55)	-6.58 (-13.32)	-2.85 (-5.77)
	MAE (ms)	11.17 ± 6.10	10.93 ± 6.38	11.02 ± 7.19	12.82 ± 6.48
	MAPE (%)	25.44 ± 14.56	20.72 ± 8.01	19.47 ± 8.21	25.20 ± 9.77
	RE (ms)	4.36 ± 5.10	5.32 ± 7.03	7.50 ± 7.94	4.70 ± 5.16
	RPE (%)	8.53 ± 9.69	9.59 ± 9.98	13.35 ± 10.36	8.92 ± 7.12

Metric results (except bias) were shown as mean ± standard deviation among the samples. Biases were calculated between the mean $T_{1\rho}$ values from ground truths and predictions within ROI. Note, ROI = region of interest, MAE = mean absolute error, MAPE = mean absolute percentage error, RE = regional error, RPE = regional percentage error, MLP = multi-layer perceptron, NLLS = non-linear least squares.

Table S5: Cartilage region metric results. I_0: $T_{1\rho}$-weighted (TSL=0), I_k: $T_{1\rho}$-weighted (TSL=30ms)

ROI	Metrics	2D U-Net (unmasked)	2D U-Net (masked)	1D MLP	NLLS
Femoral Cartilage	Bias [ms (%)]	2.36 (5.06)	-0.85 (-1.83)	-0.25 (-0.55)	-0.15 (-0.33)
	MAE (ms)	7.94 ± 6.77	5.15 ± 2.78	3.30 ± 1.10	3.54 ± 1.30
	MAPE (%)	20.15 ± 18.99	11.75 ± 6.31	7.17 ± 2.16	7.61 ± 2.46
	RE (ms)	2.49 ± 6.92	1.23 ± 2.18	0.66 ± 0.61	0.80 ± 0.63
	RPE (%)	5.65 ± 16.76	2.70 ± 5.14	1.42 ± 1.35	1.71 ± 1.36
Lateral Tibial Cartilage	Bias [ms (%)]	3.29 (8.46)	-0.53 (-1.37)	-0.59 (-1.52)	-0.57 (-1.45)
	MAE (ms)	8.43 ± 8.88	4.81 ± 2.51	3.11 ± 1.19	3.08 ± 1.01
	MAPE (%)	27.24 ± 40.29	13.04 ± 7.84	7.79 ± 2.35	7.84 ± 1.99
	RE (ms)	3.44 ± 8.90	0.93 ± 1.22	0.89 ± 0.96	0.77 ± 0.53
	RPE (%)	9.69 ± 28.75	2.43 ± 3.20	2.13 ± 1.87	1.96 ± 1.35
Medial Tibial Cartilage	Bias [ms (%)]	3.58 (8.44)	-0.64 (-1.50)	-1.28 (-3.02)	-1.03 (-2.44)
	MAE (ms)	8.99 ± 7.59	5.64 ± 2.45	3.96 ± 1.95	4.01 ± 1.78
	MAPE (%)	26.60 ± 28.71	14.26 ± 7.11	9.03 ± 3.19	9.29 ± 2.99
	RE (ms)	3.65 ± 8.26	0.97 ± 1.07	1.38 ± 1.61	1.32 ± 1.20
	RPE (%)	9.30 ± 23.53	2.36 ± 2.76	3.04 ± 2.88	3.03 ± 2.33
Patellar Cartilage	Bias [ms (%)]	2.11 (4.27)	-1.58 (-3.20)	-1.72 (-3.49)	-1.54 (-3.11)
	MAE (ms)	7.85 ± 5.88	6.14 ± 3.49	4.00 ± 2.63	4.00 ± 2.52
	MAPE (%)	19.07 ± 16.07	12.74 ± 6.21	7.52 ± 3.30	7.62 ± 3.40
	RE (ms)	2.56 ± 4.93	1.94 ± 2.75	2.03 ± 2.31	1.85 ± 1.73
	RPE (%)	5.34 ± 10.55	3.70 ± 5.32	3.68 ± 3.12	3.52 ± 2.56

Metric results (except bias) were shown as mean ± standard deviation among the samples. Biases were calculated between the mean $T_{1\rho}$ values from ground truths and predictions within ROI. Note, ROI = region of interest, MAE = mean absolute error, MAPE = mean absolute percentage error, RE = regional error, RPE = regional percentage error, MLP = multi-layer perceptron, NLLS = non-linear least squares.

Table S6: Cartilage region metric results. I_0: $T_{1\rho}$-weighted (TSL=0), I_k: $T_{1\rho}$-weighted (TSL=50ms)

ROI	Metrics	2D U-Net (unmasked)	2D U-Net (masked)	1D MLP	NLLS
Femoral Cartilage	Bias [ms (%)]	2.11 (4.53)	-0.34 (-0.73)	-0.18 (-0.39)	0.37 (0.80)
	MAE (ms)	7.81 ± 6.59	4.87 ± 2.79	2.65 ± 0.88	2.72 ± 0.90
	MAPE (%)	20.63 ± 18.56	12.11 ± 6.68	7.31 ± 3.06	7.62 ± 3.17
	RE (ms)	2.49 ± 6.67	1.00 ± 2.15	0.65 ± 0.51	0.60 ± 0.55
	RPE (%)	5.74 ± 16.17	2.29 ± 5.16	1.42 ± 1.13	1.35 ± 1.31
Lateral Tibial Cartilage	Bias [ms (%)]	3.28 (8.45)	-0.04 (-0.10)	0.70 (1.80)	0.91 (2.33)
	MAE (ms)	8.49 ± 8.67	4.78 ± 2.36	2.87 ± 0.76	2.96 ± 0.69
	MAPE (%)	27.94 ± 39.75	13.91 ± 7.48	9.41 ± 3.90	9.61 ± 3.39
	RE (ms)	3.43 ± 8.79	1.00 ± 1.26	0.86 ± 0.80	0.94 ± 0.77
	RPE (%)	9.82 ± 28.45	2.62 ± 3.32	2.39 ± 2.49	2.48 ± 2.08
Medial Tibial Cartilage	Bias [ms (%)]	3.83 (9.03)	0.31 (0.73)	0.84 (1.98)	1.34 (3.16)
	MAE (ms)	9.01 ± 7.68	5.53 ± 2.47	3.37 ± 1.06	3.53 ± 1.10
	MAPE (%)	27.92 ± 29.31	15.70 ± 8.50	10.64 ± 4.73	11.04 ± 4.51
	RE (ms)	3.84 ± 8.36	1.22 ± 1.14	1.17 ± 1.04	1.36 ± 1.09
	RPE (%)	9.82 ± 23.79	3.10 ± 3.31	3.03 ± 3.33	3.39 ± 3.06
Patellar Cartilage	Bias [ms (%)]	2.57 (5.20)	-0.29 (-0.59)	0.36 (0.72)	0.92 (1.87)
	MAE (ms)	7.53 ± 5.94	5.45 ± 3.09	2.52 ± 1.06	2.67 ± 1.00
	MAPE (%)	19.30 ± 16.43	12.62 ± 6.28	6.57 ± 2.76	7.04 ± 2.72
	RE (ms)	2.73 ± 5.07	1.34 ± 2.00	0.90 ± 0.60	1.08 ± 0.75
	RPE (%)	5.56 ± 10.82	2.76 ± 4.43	1.88 ± 1.30	2.21 ± 1.56

Metric results (except bias) were shown as mean ± standard deviation among the samples. Biases were calculated between the mean $T_{1\rho}$ values from ground truths and predictions within ROI. Note, ROI = region of interest, MAE = mean absolute error, MAPE = mean absolute percentage error, RE = regional error, RPE = regional percentage error, MLP = multi-layer perceptron, NLLS = non-linear least squares.

S4 Example $T_{1\rho}$ Predictions

Together with Figure 6 in the main text, we presented two more example $T_{1\rho}$ predictions. These slices were from two participants, one with severe OA (Figure S2), another was a healthy volunteer (Figure S3).

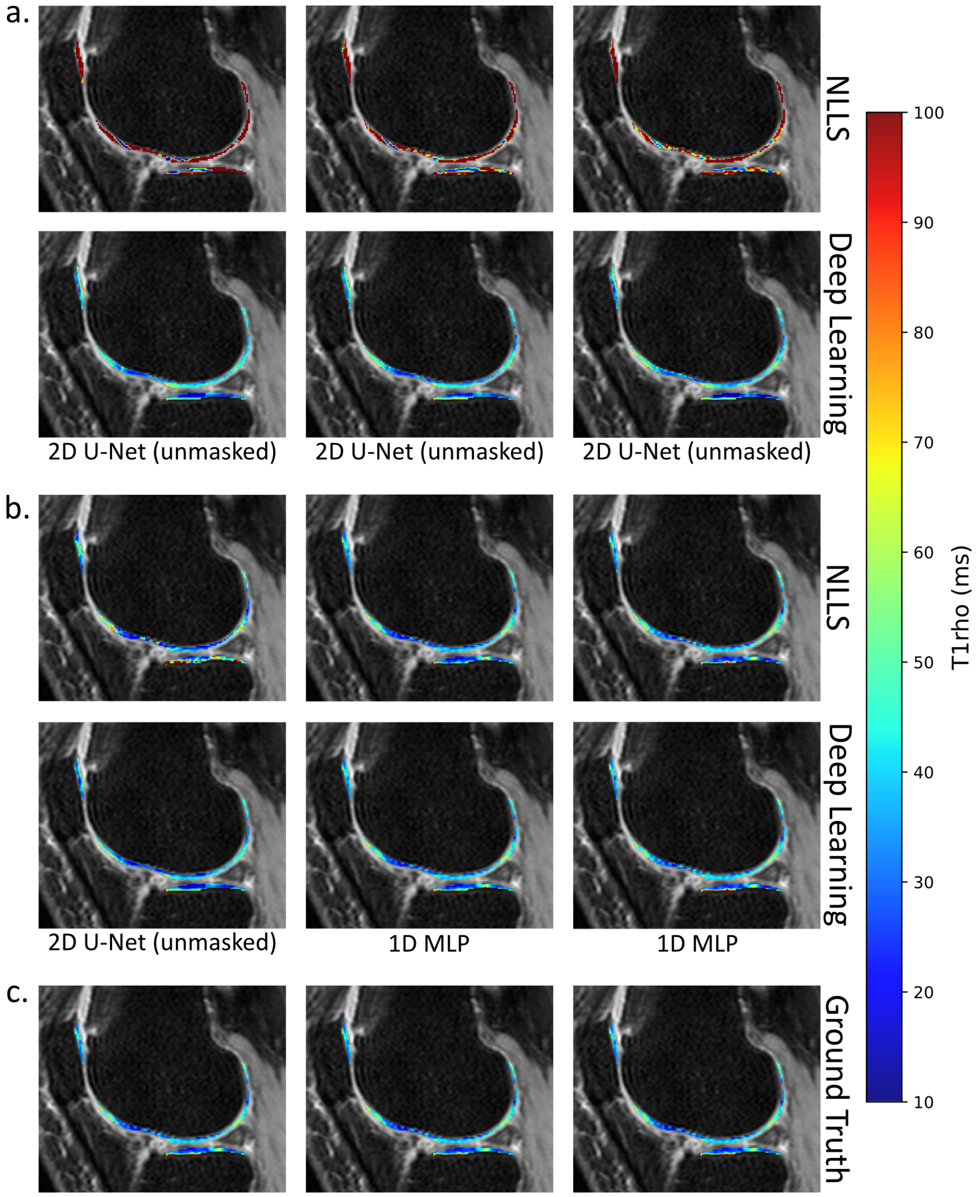

Figure S2: An example slice from a severe OA patient (74-year-old female, BMI=24.79 kg/m^2, left knee). The figure displays NLLS fitted, deep learning predicted, and ground truth $T_{1\rho}$ maps. **a.** presents $T_{1\rho}$ maps when I_0 is PD-weighted image, **b.** presents maps when I_0 is $T_{1\rho}$-weighted image, and **c.** presents the ground truth. The columns represent three different I_k settings, from left to right are $T_{1\rho}$-weighted images (TSL=10ms/30ms/50ms). Captions directly below the deep learning predictions indicate the model names. The deep learning predictions were generated from the best-performing model of each I_0-I_k combination. The three identical ground truth maps are duplicated to facilitate better interpretation of the figure. Note, NLLS=non-linear least square fitting, OA=osteoarthritis, BMI=body mass index, PD=proton density, TSL=time of spin-lock.

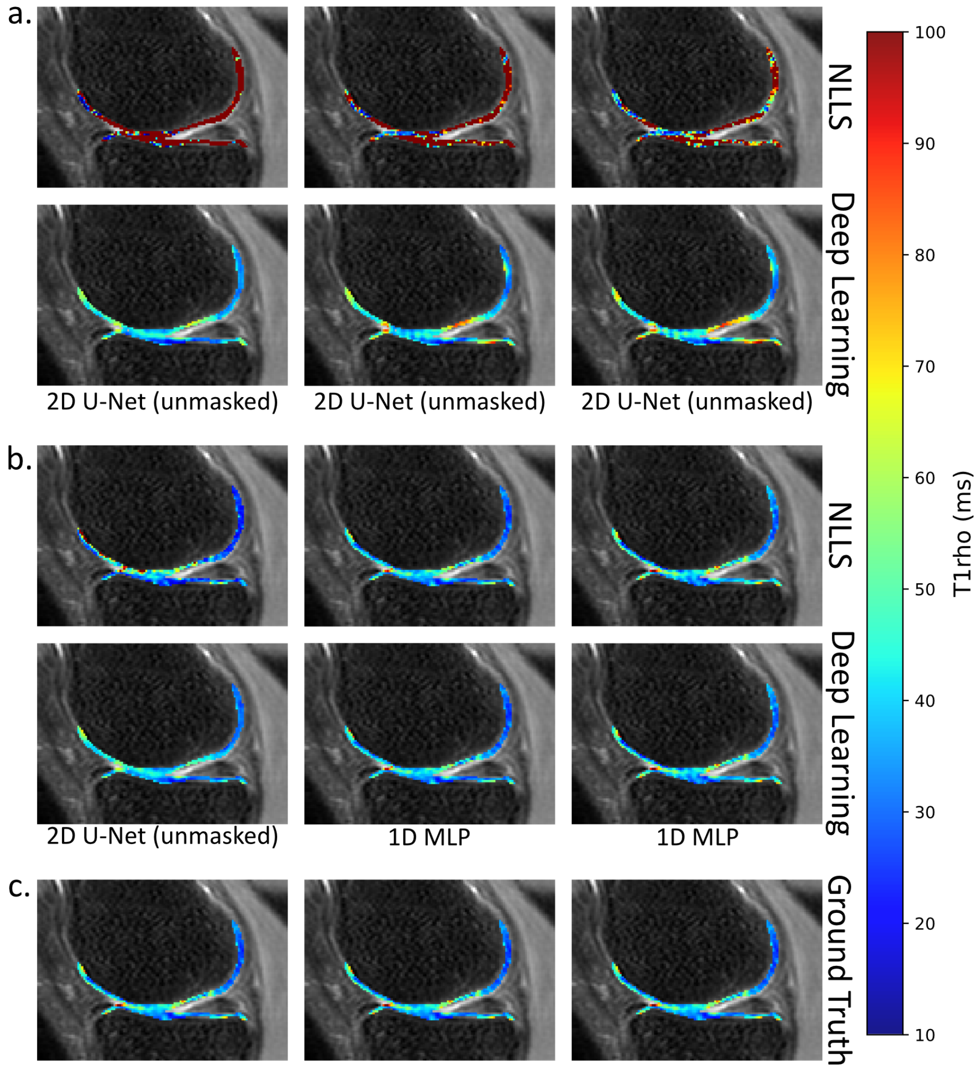

Figure S3: An example slice from a healthy volunteer (23-year-old male, BMI=20.42 kg/m^2, left knee). The figure displays NLLS fitted, deep learning predicted, and ground truth $T_{1\rho}$ maps. **a.** presents $T_{1\rho}$ maps when I_0 is PD-weighted image, **b.** presents maps when I_0 is $T_{1\rho}$-weighted image, and **c.** presents the ground truth. The columns represent three different I_k settings, from left to right are $T_{1\rho}$-weighted images (TSL=10ms/30ms/50ms). Captions directly below the deep learning predictions indicate the model names. The deep learning predictions were generated from the best-performing model of each I_0-I_k combination. The three identical ground truth maps are duplicated to facilitate better interpretation of the figure. Note, NLLS=non-linear least square fitting, OA=osteoarthritis, BMI=body mass index, PD=proton density, TSL=time of spin-lock.

References

1. Yushkevich Paul A., Piven Joseph, Cody Hazlett Heather, et al. User-guided 3D active contour segmentation of anatomical structures: Significantly improved efficiency and reliability. *Neuroimage.* 2006;31(3):1116–1128.

2. Avants B. B., Epstein C. L., Grossman M., Gee J. C.. Symmetric diffeomorphic image registration with cross-correlation: Evaluating automated labeling of elderly and neurodegenerative brain. *Medical Image Analysis.* 2008;12(1):26–41.

3. Rohlfing Torsten. Image Similarity and Tissue Overlaps as Surrogates for Image Registration Accuracy: Widely Used but Unreliable. *IEEE Transactions on Medical Imaging.* 2012;31(2):153–163.
